# TRPV1 Activation Promotes β-arrestin2 Interaction with the Ribosomal Biogenesis Machinery in the Nucleolus: Implications for p53 Regulation and Neurite Outgrowth

**DOI:** 10.3390/ijms22052280

**Published:** 2021-02-25

**Authors:** Ahmed Hassan, Mircea Iftinca, Daniel Young, Robyn Flynn, Francina Agosti, Nasser Abdullah, Manon Defaye, Mark G. H. Scott, Antoine Dufour, Christophe Altier

**Affiliations:** 1 Department of Physiology and Pharmacology, Inflammation Research Network-Snyder Institute for Chronic Diseases and Alberta Children’s Hospital Research Institute, University of Calgary, 3330 Hospital Dr NW, Calgary, AB T2N 1N4, Canada; ahmed.hassan1@ucalgary.ca (A.H.); miftinca@ucalgary.ca (M.I.); francina.agosti@ucalgary.ca (F.A.); nasser.abdullah@ucalgary.ca (N.A.); manon.defaye1@ucalgary.ca (M.D.); 2 Department of Physiology and Pharmacology, McCaig Institute for Bone and Joint Health, University of Calgary, Calgary, AB T2N 1N4, Canada; daniel.young1@ucalgary.ca (D.Y.); antoine.dufour@ucalgary.ca (A.D.); 3 Hotchkiss Brain Institute, Cumming School of Medicine, University of Calgary, Calgary, AB T2N 1N4, Canada; rflynn@ucalgary.ca; 4 INSERM-CNRS, Team: Receptor Signalling & Molecular Scaffolds, Institut Cochin, 75014 Paris, France; mark.scott@inserm.fr

**Keywords:** TRPV1, β-arrestins, ribosomal biogenesis, neuroplasticity, chronic pain

## Abstract

Transient receptor potential vanilloids (TRPV1) are non-selective cation channels that sense and transduce inflammatory pain signals. We previously reported that activation of TRPV1 induced the translocation of β-arrestin2 (ARRB2) from the cytoplasm to the nucleus, raising questions about the functional role of ARRB2 in the nucleus. Here, we determined the ARRB2 nuclear signalosome by conducting a quantitative proteomic analysis of the nucleus-sequestered L395Q ARRB2 mutant, compared to the cytosolic wild-type ARRB2 (WT ARRB2), in a heterologous expression system. We identified clusters of proteins that localize to the nucleolus and are involved in ribosomal biogenesis. Accordingly, L395Q ARRB2 or WT ARRB2 after capsaicin treatment were found to co-localize and interact with the nucleolar marker nucleophosmin (NPM1), treacle protein (TCOF1) and RNA polymerase I (POL I). We further investigated the role of nuclear ARRB2 signaling in regulating neuroplasticity. Using neuroblastoma (neuro2a) cells and dorsal root ganglia (DRG) neurons, we found that L395Q ARRB2 mutant increased POL I activity, inhibited the tumor suppressorp53 (p53) level and caused a decrease in the outgrowth of neurites. Together, our results suggest that the activation of TRPV1 promotes the ARRB2-mediated regulation of ribosomal biogenesis in the nucleolus. The ARRB2-TCOF1-p53 checkpoint signaling pathway might be involved in regulating neurite outgrowth associated with pathological pain conditions.

## 1. Introduction

Pain is a distressing sensation caused by harmful stimuli. The initiation of pain signals occurs at the periphery where noxious stimuli are detected and transduced by afferent sensory neurons that have cell soma in the dorsal root ganglia (DRG) and project centrally to the central nervous system (CNS). Acute pain is an essential physiological response to alarm against damaging insults to the body’s organs and tissues [1]. On the other hand, no physiological function is known to be associated with chronic forms of pain such as inflammatory and neuropathic pain [2]. A key property of the somatosensory nociceptive system is its plasticity. The term plasticity refers to the ability of the neuronal circuits to exhibit structural and functional adaptations to the changing physiological stimuli. Plasticity of neural pain circuits is associated with various pathological conditions including nerve injury or cancer, thus resulting in the transition from acute to chronic pain [3]. The Transient Receptor Potential Vanilloid 1 (TRPV1) is a nonselective cation channel that is a receptor for capsaicin, the irritating compound in chili pepper. It acts as a heat sensitive channel that is regulated by various noxious stimuli including low pH, changes in osmolarity, inflammatory mediators and changes in membrane potential [4]. TRPV1 is known to play a key role in regulating inflammatory pain. Sensitization of the channel by various inflammatory factors such as nerve growth factor (NGF), prostaglandins or bradykinin [5,6,7], leads to the development of thermal hyperalgesia during inflammation [8].

TRPV1 and G-protein coupled receptors (GPCRs) have been shown to regulate each other function in inflammatory pain settings. Commonly used opioids provide pain relief by activating the mu opioid receptor (MOR), which inhibits the activity of TRPV1 through the cyclic adenosine monophosphate (cAMP)/protein kinase A (PKA) and the mitogen-activated protein kinases (MAPK) pathways [9]. ARBB2 is a central signaling protein that regulates the desensitization of many GPCRs [10], including MOR. Recently, we demonstrated that the activation of TRPV1 induced the shuttling of ARRB2 to the nucleus, thus inhibiting the ARRB2-mediated desensitization and internalization of MOR [11]. This finding is important as it may explain how inflammation enhances the analgesic efficacy of opioids and support the therapeutic potential of capsaicin and other TRPV1 agonists.

Here, we sought to understand the role of ARRB2 translocation to the nucleus, following TRPV1 activation. We first showed that ARBB2 translocated to the nucleolus following TRPV1 activation. Using a quantitative proteomic analysis of WT ARRB2 and nucleus-localized ARRB2 L395Q mutant [12], we identified ARRB2 nuclear interacting proteins in human embryonic kidney (HEK293) cells. Interestingly, most of the identified proteins were constituents of the ribosomal biogenesis machinery, including NPM1, TCOF1 and POL I. We showed that nuclear translocation of ARRB2 increased the expression of 47S pre-rRNA, a product of POL I-mediated transcription and a precursor for mature rRNAs [13]. Given the functional relationship between ribosomal biogenesis, p53 and cellular differentiation [14,15,16], we then investigated whether nuclear localization of ARRB2 was able to regulate neuronal outgrowth in neuroblastoma cell lines and sensory neurons of the DRG. Our work provides evidence that nuclear ARRB2 regulates the biogenesis of ribosomes, p53 levels and neurite outgrowth. These findings highlight the relevance of targeting ARRB2 nuclear signaling to manage neuroplasticity.

## 2. Results

### 2.1. TRPV1 Induces the Translocation of ARRB2 to the Nucleolus

**Figure 1:**
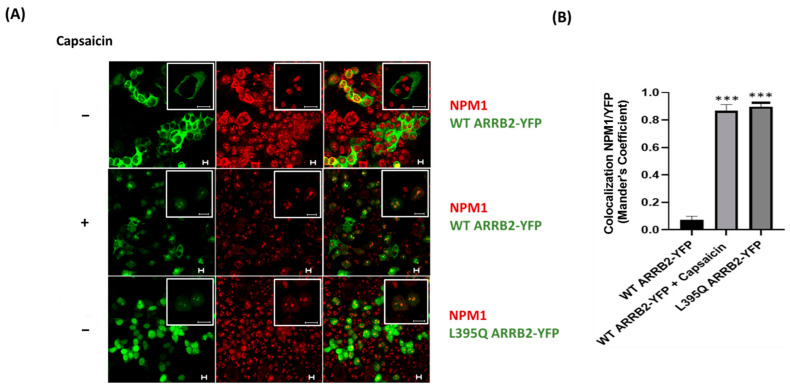
TRPV1 induces ARRB2 shuttling to the nucleolus. (**A**) Representative confocal images of co-localization between NPM1 (red) and ARRB2 (green) in HEK293 cell transfected with plasmids expressing TRPV1 and ARRB2–YFP or the NES mutant L395Q ARRB2-YFP, without or with 1 μM capsaicin for 15 min. Scale bars, 10 μm. Images are representatives of 3 independent experiments. (**B**) Quantification of the colocalization between NPM1 and YFP using the Mander’s coefficient in ImageJ. Note that NPM1 is colocalized with L395Q ARRB2-YFP mutant or WT ARRB2-YFP, after capsaicin treatment. Sample means are plotted ± SEM. Statistical analyses were performed using Kruskal–Wallis test; *** *p* < 0.001.

We previously reported that the activation of TRPV1 induced the translocation of ARRB2 into sub-nuclear structures that resembled nucleoli [11]. To determine whether ARRB2 was localized to the nucleolus, we conducted immunostaining for nucleophosmin (NPM1), an abundant nucleolar protein marker that binds single-stranded and double-stranded nucleic acids and is associated with ribonucleoprotein structures. NPM1 has been shown to regulate ribosome biogenesis, mRNA processing, chromatin remodeling, and embryogenesis [17]. In HEK293 cells transfected with TRPV1 and WT Yellow Fluorescent Protein (YFP)-tagged ARRB2, capsaicin induced the trafficking of ARRB2 in NPM1 immunopositive nucleoli (Figure 1A), as denoted by an increase in the degree of colocalization between ARRB2 and NPM1 in response to capsaicin (Figure 1B). To rule out any artefact effect of capsaicin and test the intrinsic property of ARRB2 to localize in the nucleolus, we used a mutant of ARRB2 defective of its nuclear export signal (NES), by replacing the Leucine-395 of ARRB2 with a glutamine residue. In the absence of capsaicin stimulation, the ARRB2 L395Q mutant co-localized with NPM1 in the nucleolus (Figure 1A,B), suggesting that TRPV1-mediated post-translational regulation impacts the nuclear export function of ARRB2.

### 2.2. Proteomic Analysis of ARRB2 Nuclear Interactome

**Figure 2:**
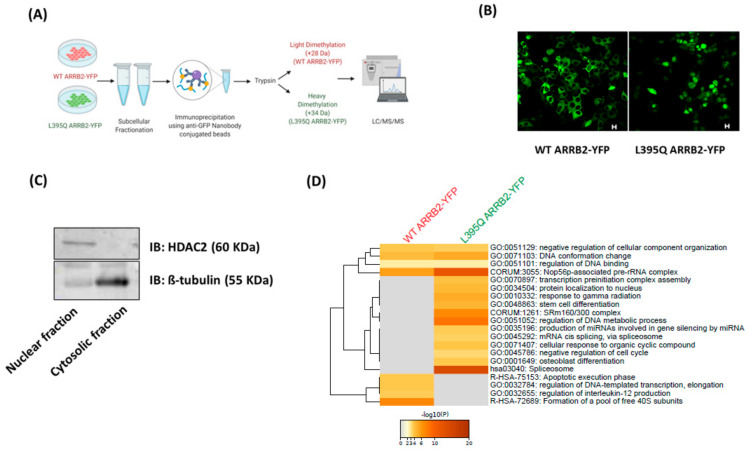
Proteomic analysis of ARRB2 nuclear interactome. (**A**) Experimental design for ARRB2 proteomic analysis. HEK293 cells were transfected with nucleus-excluded WT or nucleus-enriched L395Q ARRB2-YFP mutant. Subcellular fractionation was followed by immunoprecipitation of WT or L395Q ARRB2 protein complexes in cytosolic and nuclear fraction using anti-GFP nanobody-conjugated beads. WT and L395Q immunoprecipitated complexes were labeled with light (+28 Da) and heavy (+34 Da) formaldehyde, respectively, and mixed for LC-MS/MS analysis. Figure was created using BioRender. (**B**) Representative confocal images of HEK293 cells transfected with WT ARRB2-YFP or L395Q ARRB2-YFP used for proteomics analysis. Scale bars, 10 μm. (**C**) Representative Western blot showing the expression of the nuclear marker, HDAC2 (top panel) and the cytoskeletal marker, β-tubulin (lower panel), in the nuclear and cytosolic fractions of HEK293 cells. (**D**) Enrichment analysis by Metascape. Heat maps of enriched terms, colored by *p*-values, among the peptides upregulated in L395Q ARRB2-YFP compared to WT ARRB2-YFP protein complexes.

To determine the nuclear signalosome of ARRB2, we conducted a comparative proteomic analysis between nucleus-sequestered L395Q ARRB2-YFP mutant, immunoprecipitated from nuclear cell fractions and nucleus-excluded WT ARRB2-YFP, isolated from cytosolic cell fractions (Figure 2A–C). Proteins differentially labelled in the L395Q ARRB2 samples were identified as the nuclear interactome of ARRB2. The full list of proteins that were identified to be enriched in WT or L395Q ARRB2-YFP complexes are listed in Supplementary Tables S1 and S2. The list of proteins interacting with WT or L395Q ARRB2 were run through Metascape database (metascape.org/gp) (accessed on 17 March 2020). The analysis revealed clusters of nuclear proteins that are involved in ribonucleoprotein complexes biosynthesis and RNA splicing (Figure 2D). Among them, proteins that are part of the Nop56p-associated Pre-rRNA complex had a high score. This protein complex is involved in the biosynthesis, assembly and processing of pre-ribosomal RNA (rRNA) and the 60S ribosomal subunit. In addition to ribosomal biogenesis, ARRB2 interacting nuclear proteins were found to be involved in other cellular processes including nuclear transport, chromatin remodeling and regulation of gene transcription (Figure 2D).

### 2.3. ARRB2 Interacts with Proteins Involved in Ribosomal Biogenesis

**Figure 3:**
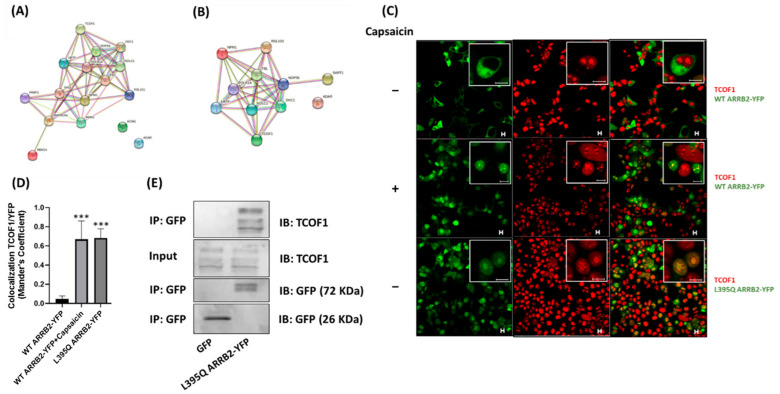
ARRB2 interacts with proteins involved in ribosomal biogenesis. (**A**) Proteins found to be enriched in L395Q ARRB2-YFP immunoprecipitated complexes were mapped against Homo Sapiens database using the STRING v11. The circles represent proteins, and the colored straight lines represent the type of evidence of protein–protein interactions; experimentally determined interactions (pink), text mining (yellow), protein homology (purple) gene neighborhood (green), co-expression (black), gene fusions (red), co-occurrence (blue), known interactions (teal). STRING analysis revealed a cluster of proteins that are components of the nucleolus. (**B**) Analysis identified a subset of the nucleolar proteins that are involved in ribonucleoprotein complex biogenesis. (**C**) Representative confocal image of colocalization between TCOF1 (red) and ARRB2 (green) in HEK293 cells transfected with plasmids expressing TRPV1 and ARRB2–YFP or the L395Q ARRB2-YFP mutant, without or with 1 μM of capsaicin for 15 min. Scale bars, 10 μm. (**D**) Quantification of the colocalization between TCOF1 and YFP using the Mander’s coefficient in ImageJ. Note that TCOF1 is colocalized with L395Q ARRB2-YFP mutant or WT ARRB2-YFP, after capsaicin treatment. Sample means are plotted ± SEM. Statistical analyses were performed using Kruskal–Wallis test; *** *p* < 0.001. (**E**) Co-immunoprecipitation analysis between TCOF1 and L395Q ARRB2-YFP or green fluorescent protein (GFP) control. Immunoprecipitates from transfected HEK293 cells were immunoblotted for TCOF1 (upper panel), input membrane immunoblotted (IB) with antibody to GFP which recognizes GFP and YFP. Molecular masses in kDa are indicated on the right. Immunoprecipitation is representative of three independent experiments. Statistical analyses were performed using Kruskal–Wallis test; *** *p* < 0.001.

To further identify the protein–protein interaction networks of ARRB2 and to gain insight into the cellular processes implicated, we used STRING v11 database analysis (https://string-db.org/) (accessed on 14 June 2019). Having demonstrated that ARRB2 translocated to the nucleolus following TRPV1 activation, and given that the nucleolus is a dynamic boundless structure where rRNA transcription, pre-rRNA processing and the assembly of ribosome subunits take place [18], we screened for ARRB2-interacting proteins involved in ribosomal biogenesis. Using the STRING analysis, we identified TCOF1, nucleolar and coiled-body phosphoprotein (NOLC1), upstream binding transcription factor (UBTF), nucleolar protein 56 (NOP56), NPM1, fibrillarin (FBL) and H/ACA ribonucleoprotein complex subunit DKC1 (DKC1) which are all related to ribosomal biogenesis and mRNA translation (Figure 3B). TCOF1 and NOLC1 associate with each other and serve as a platform to link POL I with enzymes responsible for ribosomal synthesis and modification [19]. UBTF, on the other hand, activates the transcription of rRNA by POL I [20], while NPM1 is involved in ribosome assembly [21] and in promoting the cleavage and the maturation of pre-rRNA [22]. TCOF1, along with NOLC1 and UBTF, was the most abundant protein identified in our proteomics experiment. We thus performed immunocytochemistry and co-immunoprecipitation to test the interaction between ARRB2 and TCOF1 in HEK293 cells transfected with TRPV1. As previously reported [23], TCOF1 was found in the nucleolus where it is known to be active. In the absence of capsaicin, WT ARRB2 was excluded from the nucleolus and did not colocalize with TCOF1 (Figure 3C,D). In contrast, following incubation with capsaicin, a high degree of colocalization between TCOF1 and ARRB2 or nucleus-sequestered L395Q ARRB2 mutant, was measured (Figure 3C,D). These results suggested that, when shuttled to the nucleolus, ARRB2 was able to interact with TCOF1. We then used co-immunoprecipitation to further confirm the interaction between ARRB2 and TCOF1. As the incubation of HEK293 cells with the protein lysis buffer alone was able to promote the translocation of WT ARRB2, we used the GFP plasmid as a negative control. As observed by immunostaining, ARRB2-TCOF1 interaction was confirmed biochemically following immunoprecipitation (Figure 3E).

### 2.4. ARRB2 Interacts with RNA Polymerase I

**Figure 4:**
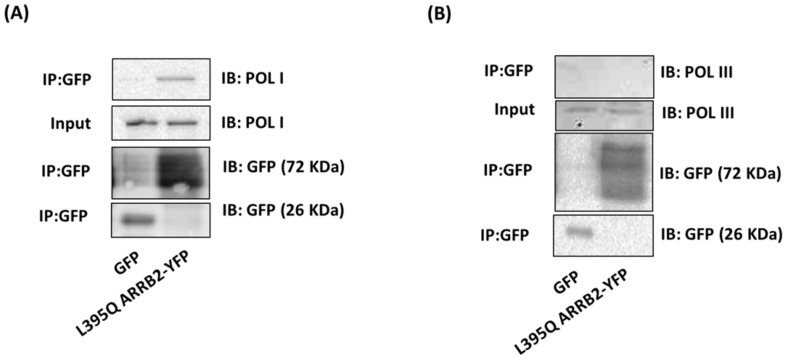
ARRB2 interacts with POL I but not POL III in the nucleus. (**A**) Co-immunoprecipitation analysis between POLI and L395Q-ARRB2-YFP or GFP protein complexes from transfected HEK293 cells. Western blot shows interaction between L395Q ARRB2-YFP and POL I (upper panel), input membrane immunoblotted (IB) with antibody to GFP which recognizes GFP and YFP. Molecular masses in kDa are indicated on the right. (**B**) Co-immunoprecipitation analysis between POL III and L395Q ARRB2-YFP or GFP protein complexes from transfected HEK293 cells, using a GFP antibody that recognizes YFP. Western blot shows absence of interaction between L395Q ARRB2-YFP and POL III (upper panel), input membrane immunoblotted (IB) with GFP antibody. Molecular weight in kDa is indicated on the right. Co-immunoprecipitations are representative of three independent experiments.

TCOF1 is involved in forming the machinery necessary for ribosomal RNA transcription, through its interaction with POL I and UBTF in the nucleolus [24]. The three RNA polymerases (I, II and III) contribute, in a coordinated manner, to the process of ribosome synthesis [25]. POL I transcribes 5.8S, 18S and 18S rRNAs that are constituents of the mature ribosomal subunits. After validating the interaction between ARRB2 and TCOF1, we tested whether POL I was part of the rRNA transcription complex. As performed in Figure 3, immunoprecipitation experiments using L395Q ARRB2-YFP or GFP control identified RNA POL I in complex with the nucleus-enriched L395Q ARRB2 (Figure 4A), indicating an interaction between ARRB2 and POL I in the nucleus. However, RNA polymerase III (POL III), another polymerase that is involved in rRNA transcription, was not found to be part of the ARRB2 signaling complex (Figure 4B).

### 2.5. ARRB2 Nuclear Translocation Decreases p53 Expression in Neuro2a Cells

**Figure 5:**
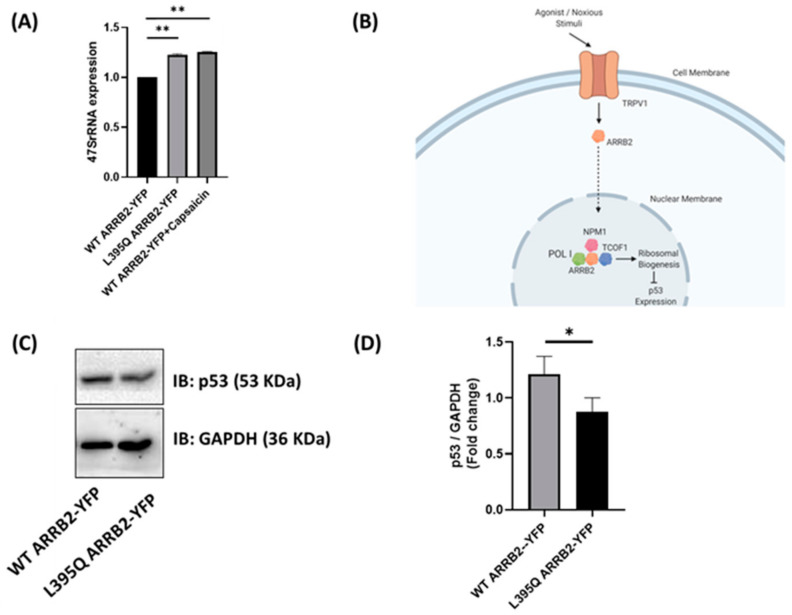
ARRB2 localization affects p53 expression. (**A**) Expression of 47S pre-rRNA, normalized to GAPDH, in neuro2a cells transfected with L395Q ARRB2-YFP or WT ARRB2-YFP with or without 1 μM capsaicin. Bar graphs represent three independent experiments. Statistical analyses were performed using Kruskal–Wallis followed by Dunn’s post hoc test; ** *p* < 0.01. (**B**) A schematic diagram of the proposed pathway for the regulation of p53 expression by ARRB2 through interaction with the ribosomal biogenesis machinery. Figure was created using BioRender. (**C**) Western blot analysis of p53 in neuro2a cells transfected with WT ARRB2-YFP or L395Q ARRB2-YFP. (**D**) Bar chart representing the protein expression level of p53 (normalized to GAPDH) illustrated in C. Statistical analyses were performed using Mann–Whitney U test; * *p* < 0.05. *N* = 4 independent experiments.

The tumor suppressor p53 is a nuclear transcription factor that promotes cell-cycle arrest in response to DNA damage or other cellular stressors [26]. TCOF1, through its role in ribosomal biogenesis, has been shown to inhibit the expression of p53 and its downstream genes—Pmaip1, Ccng1, Trp53inp1, Perp and Wig1 [27]. A reduction in p53 levels was shown to be associated with an upregulation of rRNA synthesis and ribosomal biogenesis, in response to cytotoxic stress. Donati et al. found that a decreased availability of ribosomal proteins for the E3 ubiquitin-protein ligase Mdm2 led to an increase in MDM2-mediated degradation of p53 [14]. In addition to its well known pro-apoptotic effects, p53 has been shown to promote neuronal differentiation [16], including neuronal outgrowth and branching, which are processes that are associated with changes in neuronal excitability that contributes to the development of chronic pain [28]. We thus assessed the influence of ARRB2 shuttling on rRNA synthesis and p53 levels. Expression of 47S pre-rRNA—the precursor for mature rRNAs 18S, 5.8S, and 28S—was enhanced in neuro2a cells transfected with the L395Q ARRB2-YFP mutant, or after capsaicin activation of TRPV1 (Figure 5A). As p53 is regulated by ribosomal biogenesis (Figure 5B), we next sought to examine whether ARRB2-induced increase in rRNA synthesis could alter p53 level and neurite outgrowth.

Using Western blot analysis, we found that the mutant of ARRB2 interacting with the ribosomal biogenesis machinery was able to lower p53 levels, when compared to WT ARRB2 (Figure 5C,D). These results suggest that ARRB2 interaction with proteins involved in ribosomal biogenesis including NPM1, TCOF1 and POL I, induced down regulation of p53.

### 2.6. ARRB2 Nuclear Localization Decreases Neurite Outgrowth in Neuro2a Cells

**Figure 6:**
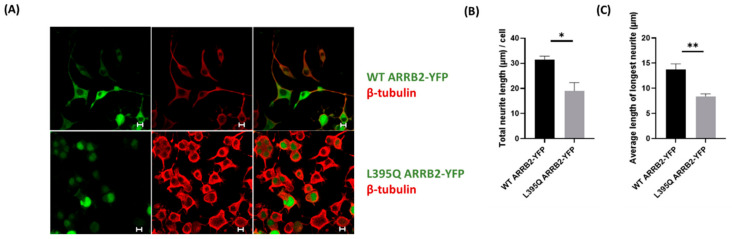
ARRB2 nuclear localization decreased neurite outgrowth in neuro2a cells. (**A**) Representative confocal images of neuro2a cells transfected with WT ARRB2-YFP or L395Q ARRB2-YFP. Neurite branching and length were determined using ß-tubulin immunostaining and quantification by ImageJ (**B**,**C**). Scale bars, 10 μm. * *p* < 0.05; ** *p* < 0.01 by Mann–Whitney U test. *N* = 30 images analyzed from three independent experiments.

Following the observation that ARRB2 nuclear localization caused a decrease in p53 expression, we sought to examine the effect of ARRB2-induced p53 suppression on neurite outgrowth. Neuro2a cells were transfected with either WT or L395Q ARRB2-YFP. As observed in the transfected HEK293 cells, the L395Q ARRB2 mutant was sequestered in the cell nucleus compared to WT ARRB2 (Figure 6A). To induce differentiation, neuro2a cells were serum-starved for 24 h post-transfection, before β-tubulin immunostaining. As shown in Figure 6A, serum starvation promoted mitotic arrest and the extension of several neurites per cell. Analysis of the neurite outgrowth and branching revealed that nuclear accumulation of the L395Q ARRB2 mutant induced an overall decrease in neurite growth and the average length of the longest neurite/cell, compared to WT ARRB2-YFP (Figure 6B,C).

### 2.7. ARRB2 Nuclear Localization Decreases Neurite Outgrowth in DRGs

**Figure 7:**
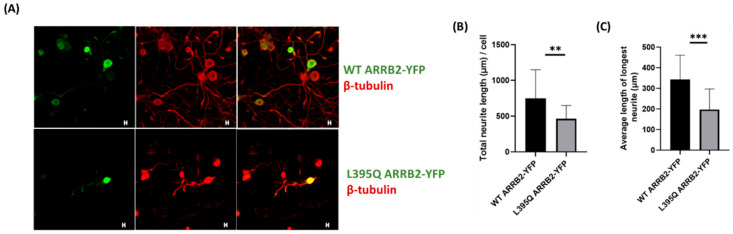
ARRB2 nuclear localization decreases neurite outgrowth in DRG neurons. (**A**) Representative confocal image of cultured DRG neurons transduced with WT ARRB2-YFP or L395Q ARRB2-YFP, and immunostained for ß-tubulin. Neurite branching and length were determined using ß-tubulin immunostaining and quantification by ImageJ (**B**,**C**). Scale bars, 10 μm; ** *p* < 0.01; *** *p* < 0.001 by Mann–Whitney U test. *N* = 40 images analyzed from three independent experiments.

We then aimed to investigate if nuclear sequestration of ARRB2 would affect neurite outgrowth in isolated DRG neurons. Dissociated neurons were infected with adeno associated virus (AAV) expressing either WT ARRB2-YFP or L395Q ARRB2-YFP. Seventy-two hours following viral transduction, neurite outgrowth was assessed (Figure 7A). We found that, similar to what was observed in HEK293 and Neuro2a, L395Q ARRB2 mutant was sequestered in the cell nucleus compared to WT ARRB2 (Figure 7A). Consequently, viral transduction of L395Q ARRB2-YFP induced a decrease in both the total neurite outgrowth and average length of the longest neurite/neuron, compared to WT ARRB2-YFP-infected neurons (Figure 7B,C). These results indicate that ARRB2 nuclear interactions promote neuroplasticity of primary afferent sensory neurons, which could be implicated in the transduction and transmission of pain signals.

### 2.8. Inhibition of RNA POL I Activity Induces Neurite Outgrowth of Neuro2a Cells and DRG Neurons

**Figure 8:**
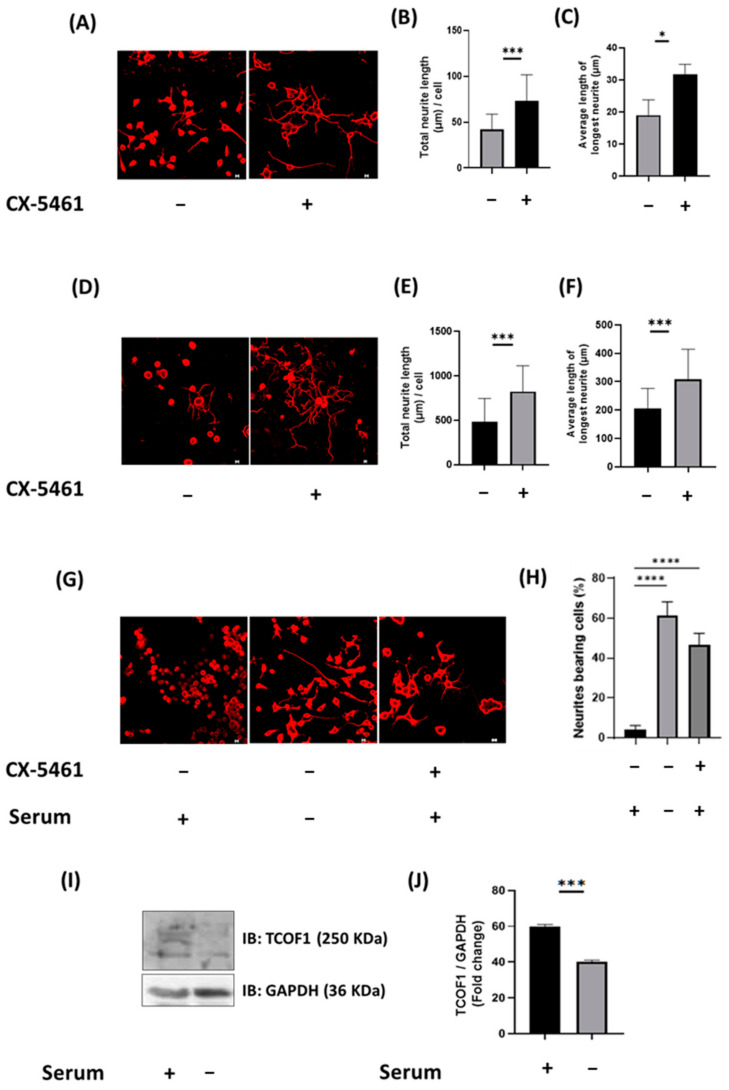
Inhibition of POL I activity enhanced neurite outgrowth in neuro2a cells and DRG neurons. (**A**) Representative confocal images of serum-starved neuro2a cells, 24 h following treatment with 100 nM CX-5461. Scale bars, 10 μm. ß-tubulin immunostaining was used to determine neurite branching and length. Quantification was done using ImageJ (**B**,**C**). (**D**) Representative confocal images of cultured DRG neurons 24 h post-treatment with CX-5461. Total neurite outgrowth and the average length of the longest neurite were quantified using ImageJ (**E**,**F**). (**G**) Representative confocal images of neuro2a cells with or without serum and treated with 100 nM CX-5461. The percentages of cells with neurites were determined and depicted on the bar graphs (**H**). Statistical analysis was performed using Kruskal–Wallis followed by Dunn’s post hoc test. (**I**) Western blot analysis of TCOF1 in serum-starved differentiated vs. undifferentiated neuro2a cells. (**J**) Bar chart representing the protein level of TCOF1 (normalized to GAPDH) illustrated in I. Statistical analysis was performed using Mann–Whitney U test; * *p* < 0.05;*** *p* < 0.001; **** *p* < 0.0001. *N* = 3 independent experiments.

To investigate the link between ribosomal biogenesis and neurite outgrowth, we used the CX-5461 compound, a highly selective inhibitor of POL I transcription [29]. At 100 nM, CX-5461 induced a significant increase in neurite outgrowth in both differentiated neuro2a cells (Figure 8A–C) and cultured DRG neurons (Figure 8D–F) 24 h post-treatment. Notably, CX-5461 alone could induce neurite outgrowth in neuro2a, regardless of the presence of serum (Figure 8G,H), suggesting that the inhibition of ribosomal biogenesis is sufficient to promote neuronal differentiation and neurite outgrowth. Furthermore, following serum starvation, differentiated neuro2a cells exhibiting neurite extension expressed less TCOF1 protein, compared to undifferentiated cells (Figure 8I,J). These data demonstrate that inhibition of ribosomal biogenesis promotes neurite outgrowth, and identified that the ARRB2-interacting protein, TCOF1, exerts a putative inhibitory role on neuronal differentiation.

## 3. Discussion

We previously reported that the activation of the TRPV1 channel leads to the shuttling of ARRB2 from the cytosol to the nucleus [11]. Here, we propose a role for the TRPV1-ARRB2 signaling axis in regulating neuroplasticity through the interaction between ARRB2 and elements of the ribosomal biogenesis machinery in the nucleolus.

TRPV1 is a key transducer of inflammatory pain. Following infection or injury, TRPV1 activity is sensitized by various pro-inflammatory mediators such as nerve growth factor (NGF), prostaglandins and bradykinin [6,7,8], which then leads to thermal hyperalgesia [9]. Importantly, inflammation primes the analgesic effects of opioids, and we showed that TRPV1 participates in enhancing opioid analgesic effects in mice by inducing the translocation of ARRB2 from the cytoplasm to the nucleus, thus preventing the subsequent ARRB2-mediated internalization and desensitization of the receptor. The observed TRPV1-induced nuclear translocation of ARRB2 raised questions regarding the role of ARRB2 in the nucleus of afferent sensory neurons.

Here, we identified ARRB2 nuclear-interacting proteins by using a proteomic analysis approach in heterologous expression systems. To maximize the expression of ARRB2 in the nucleus and overcome the limitation of transfection efficiency when co-expressing TRPV1 and ARRB2, we took advantage of a nucleus-sequestered ARRB2 mutant with a point mutation in the 395-leucine residue in its NES. The L395Q ARRB2 allowed us to mirror the effect of TRPV1-induced ARRB2 nuclear translocation, while obtaining a high enough expression of ARRB2 in the nucleus (Figure 2B). To specifically identify and quantify ARRB2 signalosomes in the cytoplasm and the nucleus, we performed immunoprecipitation of recombinant WT and mutant ARRB2 on nuclear versus cytoplasmic fractions and compared the interacting protein enrichment. Using Metascape and STRING analysis, we were able to get insights into the network of protein–protein interactions in the nucleus and their corresponding cellular processes. Interestingly, the Metascape analysis revealed the enrichment of proteins that are parts of the Nop56p-associated Pre-Ribosomal Ribonucleoprotein complexes. Notably, Nop56p is a nucleolar protein that forms a complex with Nop58p and fibrillarin and is involved in ribosomal assembly and pre-rRNA processing [30]. Analysis of our data using the STRING database revealed that most of the identified proteins interacting with ARRB2 in the nucleus are components of the nucleolus, including NPM1, MDM2 and FBL. A co-localization analysis of NPM1 and ARRB2-YFP following TRPV1 activation or when using the nucleus sequestered L395Q ARRB2 confirmed the presence of ARRB2 in the nucleolus. Remarkably, the STRING analysis points to most of the nucleolar proteins obtained in our proteomics study, such as TCOF1, POL I, UBTF and NPM1, which all participate in ribosomal biogenesis.

Ribosomes represent the core component of the translation machinery, where mRNAs are translated into proteins. The protein translational activity plays a central role in regulating long-term plasticity in afferent sensory neurons in response to various stress conditions including inflammation or injuries [31,32,33]. This contributes to phenotypic changes underlying nociceptor sensitization and the development of chronic pain. Maladaptive structural and functional alterations in neurons such as structural reorganization or changes in action potential firing are associated with de novo protein synthesis [34]. Key proteins that are synthesized in response to cellular stress include cytoskeletal proteins that are involved in neurite outgrowth and the branching of dendritic tree or proteins that regulate intrinsic excitability and neurotransmission, such as voltage-gated calcium and sodium channels [35].

An abundant protein identified in our screen was TCOF1, which is a nucleolar phosphoprotein that is involved in ribosomal biogenesis. Using immunocytochemistry and co-immunoprecipitation experiments, we confirmed the mass spectrometry results and revealed that ARRB2 and TCOF1 are part of a unique macromolecular complex that most likely regulates POL I within the nucleolus. This interaction may thus indicate a role for ARRB2 in regulating TCOF1 function. TCOF1 forms a complex with the NOLC1 [36]. This complex connects POL I with the box H/ACA ribonucleoproteins that promote rRNA pseudouridylation and the assembly and maturation of the small subunit of eukaryotic ribosomes. As recent findings predict that TCOF1 encodes a highly phosphorylated nucleolar protein [37], it remains to be seen whether translocation of ARRB2 promotes the phosphorylation of TCOF1 and thus its signaling to the ribosomal biogenesis machinery. Finally, our results do not address the role of ribosomal biogenesis and TCOF1 in inflammatory pain signaling. However, using the complete Freund’s adjuvant (CFA) model of inflammatory pain, we observed an upregulation of TCOF1 by immunohistochemistry and Western blot analysis in lumbar DRGs (Supplementary Figure S1). Overall, our work points to TCOF1 as a putative novel regulator of nociceptor sensitization in the context of tissue inflammation.

The critical regulators of ribosomal biogenesis POL I and POL III, which are responsible for transcribing rRNAs, are major constituents of the mature ribosome. We used co-immunoprecipitation experiments to test the interaction of ARRB2 with POL I and POL III. Our results showed that ARRB2 only interacts with POL I. Ribosomal biogenesis is known to be the rate limiting step in protein synthesis. Local protein synthesis in dendrites or axons are mechanisms through which neurons can respond to environmental cues, including stressors, damaging molecules and inflammatory mediators [38]. Thus, ARRB2 may be involved in inflammatory pain through regulating ribosome biogenesis and protein synthesis, two key processes underlying neuroplasticity.

Searching for potential targets of ARRB2 interactors, we identified p53. In TCOF1 haploinsufficient embryos, there is an increase in p53 expression and activity, likely due to deficiency in ribosome biogenesis, which leads to pro-apoptotic gene expression, such as Wig1, Trp53inp1 and Ccng in the neural crest [15]. In addition, increase in rRNA transcription and ribosomal biogenesis have been shown to cause a decrease in p53 levels in cancer cells [14]. We hypothesized that the interaction between ARRB2 and the ribosomal biogenesis machinery could affect p53 expression. The tumor suppressor p53 is a transcription factor that is known for its pro-apoptotic activity. In addition to its role in apoptosis, p53 has been shown to promote neuronal differentiation and neurite outgrowth [39]. These effects are mediated by the two transcriptional targets of p53, the actin-binding protein Coronin 1b and the GTPase RAB13, which associate with the cytoskeleton and promote neurite outgrowth [16]. Since structural remodeling and synaptic reorganization contribute to nociceptive sensitization and the development of chronic pain [28], we investigated the role ARRB2 in regulating p53 expression and neurite outgrowth. In neuro2a cells, ARRB2 shuttling to the nucleus resulted in an increase in 47S pre-rRNA expression, a product of POL I and an essential factor in ribosomal biogenesis. This was associated with a decrease in p53 and a reduction in neurite outgrowth. To assess the functional link between ribosomal biogenesis and neurite outgrowth, we tested the effect of POL I inhibition. We found that inhibiting POL I transcriptional activity resulted in enhancing neurite outgrowth in neuro2a cells and DRG neurons. Interestingly, neurite elongation was linked to TCOF1 expression, suggesting a negative regulatory role of TCOF1 in neuronal differentiation. These results support previous work showing that TCOF1 is required for the formation and proliferation of neural crest cells, via controlling the production of mature ribosomes [40,41].

We thus propose a model in which ARRB2 nuclear signaling governs dendritic or axon terminal arborization. Overall, our work highlights the role of ARRB2 in regulating the morphology and phenotypic changes of DRG neurons, in response to inflammation, which together could promote neuronal sensitization.

## 4. Materials and Methods

### 4.1. Cell Culture and Transfection

HEK293 cells were cultured in Dulbecco’s modified Eagle’s medium (DMEM) (Invitrogen, Mississauga, ON, Canada) and 10% fetal bovine serum (FBS) (Invitrogen, Mississauga, ON, Canada). Cells were incubated in 37 °C and 5% CO_2_. Cells were seeded at ~10–20% confluency and transfected 8 h later. Cells in 60 mm dishes were transfected using the calcium phosphate (Ca_3_(PO_4_)_2_) transfection method with 1.5 and/or 4 μg of ARRB2-YFP and TRPV1 pcDNA, respectively. Experiments were conducted 40 h after transfection.

Neuro2a cells were used for neurite outgrowth assays. Cells were maintained in DMEM supplemented with 10% FBS, L-glutamine and penicillin/streptomycin (Invitrogen, Mississauga, ON, Canada). Cells were seeded at ~50% confluency and transfected 8 h later using lipofectamine 2000 (Invitrogen, Mississauga, ON, Canada) according to the manufacturer’s recommendations. For a 35 mm dish 1 μg of WT or L395Q ARRB2-YFP pcDNA and/or 1.5 μg TRPV1 pcDNA were transfected. Cell differentiation was induced 24 h later by replacing the growth medium with serum-free DMEM medium. Experiments were conducted 24 h later.

### 4.2. Mice

Six-week-old wild-type C57BL/6 mice were purchased from Jackson Laboratory (Sacramento, CA, USA). Mice were housed with 12 h light/dark cycles and under standard conditions with drinking water and food available. Experiments were conducted under protocols approved by the University of Calgary Animal Care Committee (protocol AC16-0012, approved 01/15/2019) and according to the guidelines of the Canadian Council on Animal Care.

### 4.3. CFA-Induced Inflammation

Mice received intra-plantar injections of 20 μL of CFA or saline in the right hind paw. Mice were euthanized 24 h following injection and lumbar DRGs were collected for Western blot analysis and immunostaining experiments.

### 4.4. Isolation of DRG Neurons

DRG neurons were excised from six-week-old mice and dissociated in HBSS containing 2 mg/mL collagenase and 4 mg/mL dispase for 45 min at 37 °C. DRGs were cultured at 37 °C with 5% CO_2_ and 96% humidity in DMEM supplemented with 10% FBS, 100 μg/mL streptomycin, 100 U/mL penicillin, and 100 ng/mL nerve growth factor. Cultured DRG neurons were infected with AAV constructs encoding WT or L395Q ARRB2-YFP. Viral particles (1 × 10^8^ TU/mL) were added to the culture medium (1 μL/mL) and left for 72 h before immunostaining. The AAV constructs were obtained from the Molecular Core facility in the Hotchkiss Brain Institute, University of Calgary.

### 4.5. Chemicals and Drugs

NPM1, TCOF1 and p53 anti-rabbit antibodies were obtained from Proteintech (Rosemont, IL, USA). GFP antibody was purchased from Chromotek (Munich, Germany). POL I anti-rabbit antibody and GAPDH anti-mouse antibody were obtained from Santa Cruz (Dallas, TX, USA). The TRPV1 agonist capsaicin was purchased from Sigma-Aldrich (Oakville, ON, Canada). Secondary anti-rabbit and anti-mouse horseradish peroxidase (HRP) conjugated antibodies for Western blotting were obtained from GE Healthcare (Mississauga, ON, Canada). Goat anti-rabbit alexa fluor 555 and 633 conjugated secondary antibodies for immunofluorescence staining were obtained from Molecular Probes (Mississauga, ON, Canada). CX-5461 and CFA were purchased from Sigma-Aldrich (Oakville, ON, Canada).

### 4.6. Plasmids

ARRB2 sequence was cloned into sticky-ended annealed oligonucleotides coding for YFP to generate ARRB2-YFP. L395Q ARRB2-YFP was generated by replacing the Leucine-395 residue of ARRB2 in ARRB2-YFP pcDNA with a glutamine residue as described previously [12].

### 4.7. Subcellular Fractionation

Nuclear and cytoplasmic protein fractions were extracted from HEK293 cells using the NE-PER nuclear and cytoplasmic subcellular fractionation kit (Thermo Fisher Scientific Mississauga, ON, Canada) according to the manufacturer’s recommendation. Briefly, cells were harvested in ice-cold PBS. Cells were then resuspended and lysed in the provided hypotonic buffer for 10 min on ice. The homogenates were centrifuged, and the supernatant containing the cytoplasmic proteins was collected. The remaining pellets were resuspended in nuclear extraction buffer and incubated on ice for 40 min and vortexed repeatedly at 10 min intervals. Homogenates were then centrifuged at maximum speed for 10 min and the supernatant containing the nuclear proteins was collected.

### 4.8. Co-Immunoprecipitation of ARRB2

Cell lysates were prepared as described above. Lysates were added to GFP-Trap magnetic beads (agarose beads coupled to anti-GFP VHH Nanobody) and left on a rotator at 4 °C overnight. Flow-through samples were then separated, and the beads were washed three times (5 min each) in RIPA buffer. For eluting the immunoprecipitated proteins, beads were resuspended in 30 μL RIPA+10 μL laemmli loading buffer and kept at 37 °C for 30 min. Proteins diluted in laemmli buffer were separated from beads and then run on SDS-PAGE gels.

### 4.9. Western Blot Assay

Forty-eight hours post-transfection, cells were harvested and lysed in RIPA buffer supplemented with 1% protease and phosphatase inhibitor cocktail from Thermo Fisher Scientific (Mississauga, ON, Canada). Cell lysates were centrifuged at maximum velocity for 15 min, pellets were discarded, and supernatants containing protein lysates were collected. Protein concentration was quantified by colorimetric Braford protein assay kit (Bio-Rad, Montreal, QC, Canada). Proteins were run on SDS-PAGE gel and transferred to nitrocellulose membranes. Membranes were blocked in 5% milk in tris-buffered saline (TBS-T) and then incubated overnight at 4 °C with primary antibodies (1:1000 dilution). The next day, membranes were washed and incubated with horseradish peroxidase (HRP)-conjugated secondary antibodies. Membranes were developed using an ECL chemiluminescent kit (GE Life Sciences, Mississauga, ON, Canada) and images were obtained using a Bio-Rad Chemidoc imaging system.

### 4.10. Proteomics Analysis

Nuclear and cytosolic cell fractions were isolated from HEK293 cells expressing either WT or L395Q ARRB2-YFP. To avoid nonspecific protein binding, samples were precleared with magnetic agarose beads on a rotator for 1 h at 4 °C before co-immunoprecipitation. ARRB2 complex were immunoprecipitated using magnetic GFP-Trap beads as described above. This was followed by incubation with acetone to precipitate proteins from the solution with heavy detergents. Samples were prepared for proteomics analysis using the filter-aided sample preparation protocol (FASP); protein samples were solubilized with 8 M urea in pH8 100 mM Tris-HCl. The proteins were reduced with 10 mM dithiothreitol (DTT) (Sigma, Oakville, ON, Canada) at 37 °C for 30 min and then transferred to a 30 kDa cut-off Amicon spin column (Millipore, Etobicoke, ON, Canada). Samples were alkylated with 10 mM iodoacetamide (IAA) (Sigma, Mississauga, ON, Canada) for 20 min at room temperature in the dark. Next, samples were washed with urea-tris and then with 50 mM ammonium bicarbonate solution; this was followed by digestion with trypsin (Promega, Madison, WI, USA) overnight. The samples, which needed to be compared in pairs, were mixed with 22 μL of 20 mM deuterated formaldehyde (heavy formaldehyde) (+34.063116 Da) or 22 μL of 20 mM regular formaldehyde (light formaldehyde) (+28.031300 Da), followed by 22uL 10mM sodium cyanoborohydride for every sample. The pH was adjusted to 6.5 and samples were incubated at 37 °C for 3 h. Sample pairs with light and heavy formaldehyde labelling were then mixed and desalted with a C18 Sep-Pak cartridge (Waters, Mississauga, ON, Canada). Next, peptides were dried in a SpeedVac and dissolved in 0.1% trifluoroacetic acid (TFA) prior to LC−MS/MS. Peptides were matched to spectral data in the human UniProt protein database using MaxQuant software v.1.6.0.1 and implementing the Andromeda algorithm at a false discovery rate (FDR) of 5% for peptide-spectrum match. Pathways analyses were performed by identifying the gene ontology (GO) terms that were statically enriched with proteins that are differentially abundant between samples and controls using Metascape, with Homo Sapiens as the model organism at an FDR of 1%. The protein–protein interactions were identified using the STRING v11 database.

### 4.11. Immunocytochemistry

HEK293, neuro2a cells and dissociated DRG neurons were cultured on glass coverslips previously treated with HBSS + 25% poly-L-ornithine and laminin (Sigma, Oakville, ON, Canada) and placed on either 35 mm dishes or a 24-well plate. Before immunostaining, cells were washed twice and fixed with 4% paraformaldehyde (PFA) for 15 min at 37 °C. Next, cells were permeabilized by adding 5% bovine serum albumin (BSA) (Sigma, Oakville, ON, Canada) with 0.3% Triton X-100 (Sigma, Oakville, ON, Canada) for 1 h at room temperature. Cells were incubated overnight with primary antibody in 1% BSA with 0.01% Triton X-100. On the next day, cells were washed three times, for 20 min each, with PBS and then incubated with the secondary antibody for 1 h at room temperature. Cells were washed for 30 min in PBS and imaged using a Zeiss LSM-510 Meta inverted microscope.

### 4.12. Immunohistochemistry

Lumbar DRGs were harvested from euthanized C57BL/6 mice. DRGs were then fixed with 4% PFA and incubated in 30% sucrose for 24 h at 4 °C. DRGs were then embedded in optimal cutting temperature solution (Thermo Fisher scientific, Mississauga, ON, Canada). Embedded tissues were cut into 10-μm-thick slices and mounted on Superfrost Plus slides (VWR, Edmonton, AB, Canada) with Aqua PolyMount (Polysciences, Warrington, PA, USA). After fixation, tissues were blocked and permeabilized by incubation in 5% BSA with 0.3% Triton X-100 for 1 h. Samples were then washed and incubated with primary antibody in 1% BSA with 0.01% Triton X-100 for 24 h at 4 °C. Next, samples were washed and incubated with secondary antibody in 3% BSA with 0.01% Triton X-100 for 1 h at room temperature. Samples were then washed in PBS before confocal imaging.

### 4.13. qPCR

Neuro2a cells were homogenized in RLT buffer (Qiagen, Toronto, ON, Canada). Total RNA was extracted using a RNeasy Mini kit (Qiagen, Toronto, ON, Canada), according to the manufacturer’s instructions. The quality and quantity of RNA were determined using a Nanodrop 2000c spectrophotometer (Thermo Fisher Scientific, Mississauga, ON, Canada). cDNA was synthesized by reverse transcription using MultiScribe™ Reverse Transcriptase (Thermo Fisher Scientific, Mississauga, ON, Canada) and qPCR was performed using BrightGreen 2X qPCR MasterMix-ROX (Applied Biological Materials, Richmond, BC, Canada) and a StepOnePlus real-time PCR-detection system (Applied Biosystems, Mississauga, ON, Canada). The following primers were used: 47S-5′ETS—5′-ACACGCTGTCCTTTCCCTATTAACACTAAA-3’ and 5′-AGTAAAAAGAATAGGCTGGACAAGCAAAAC-3′ [42]; GAPDH—5′-GATGCTGGTGCTGAGTATGTCG-3’ and 5′-GTGGTGCAGGATGCATTGCTGA-3’.

### 4.14. Statistical Analysis

Data analysis was done using GraphPad Prism 7 (GraphPad software). Results were expressed as means ± S.E.M. Statistical analyses were completed with unpaired t-tests followed by the Mann–Whitney U test when comparing two means. The Kruskal–Wallis test was used when comparing two conditions to the same control. The statistical significance cut-off was set at *p* < 0.05.

## 5. Conclusions

Here, we examined the nuclear interactome of ARRB2 and identified nucleolar proteins that link ARRB2 signaling to ribosomal biogenesis. Our results identified ARRB2 interacting proteins in the nucleolus and their potential role in regulating neurite outgrowth and branching. Our work suggests a model in which ARRB2 interacts with a macromolecular complex formed by NPM1, TCOF1 and POL I to enhance ribosomal biogenesis and reduce p53 expression. This process, in turn, may lead to a decrease in neurite elongation. Thus, an ARRB2 nuclear signaling pathway may govern the neuroplasticity of primary afferent neurons underlying adaptation to danger signals including pathogen products or chemical irritants that activate the nociceptive TRPV1 channel.

## Data Availability

Data is contained within the article or Supplementary Material.

## Supplementary Materials

The following are available online at https://www.mdpi.com/1422-0067/22/5/2280/s1, Table S1: proteins interacting with L395Q ARRB2-YFP, Table S2: proteins interacting with WT ARRB2-YFP, Figure S1: TCOF1 protein level is upregulated in lumbar DRGs, 24 h following CFA injection.

## Abbreviations

ARRB2	β-arrestin2
BSA	Bovine serum albumin
cAMP	Cyclic adenosine monophosphate
Ccng1	Cyclin G1
DMEM	Dulbecco’s Modified Eagle’s Medium
DRG	Dorsal root ganglia
DTT	Dithiothreitol
DKC1	H/ACA ribonucleoprotein complex subunit DKC1
FASP	filter-aided sample preparation protocol
FBS	Fetal bovine serum
GFP	Green Fluorescent protein
HBSS	Hank’s Balanced Salt Solution
HEBS	HEPES-buffered saline solution
HEK293	Human embryonic kidney cells
IAA	Iodoacetamide
LC−MS/MS	liquid chromatography and tandem mass spectrometry
NES	Nuclear export signal
neuro2a	Mouse neuroblastoma cells
NGF	Nerve growth factor
NOLC1	Nucleolar and coiled-body phosphoprotein 1
NOP56	Nucleolar protein 56
NPM1	Nucleophosmin 1
p53	Tumor protein p53
PARP1	Poly [ADP-ribose] polymerase 1
PBS	Phosphate-Buffered Saline
PKC	protein kinase C
PKRDC	DNA-dependent protein kinase catalytic
Pmaip1	Phorbol-12-Myristate-13-Acetate-Induced Protein 1
rRNA	Ribosomal RNA
POL I	RNA Polymerase I
POL III	RNA Polymerase III
TBS-T	Tris-buffered saline
TCOF1	Treacle Ribosome Biogenesis Factor 1
TRPV	Transient Receptor Potential cation channel, subfamily V (vanilloid)
Wig1	wild-type p53-induced gene 1
YFP	Yellow fluorescent protein
