# Supplementary material for: TRPV1 Activation Promotes β-arrestin2 Interaction with the Ribosomal Biogenesis Machinery in the Nucleolus: Implications for p53 Regulation and Neurite Outgrowth
Source: Int J Mol Sci. 2021 Feb 25;22(5):2280. doi: 10.3390/ijms22052280 (PMC7956682; doi:10.3390/ijms22052280)
Supplement: Supplementary file 1 [file ijms-22-02280-s001.pdf]

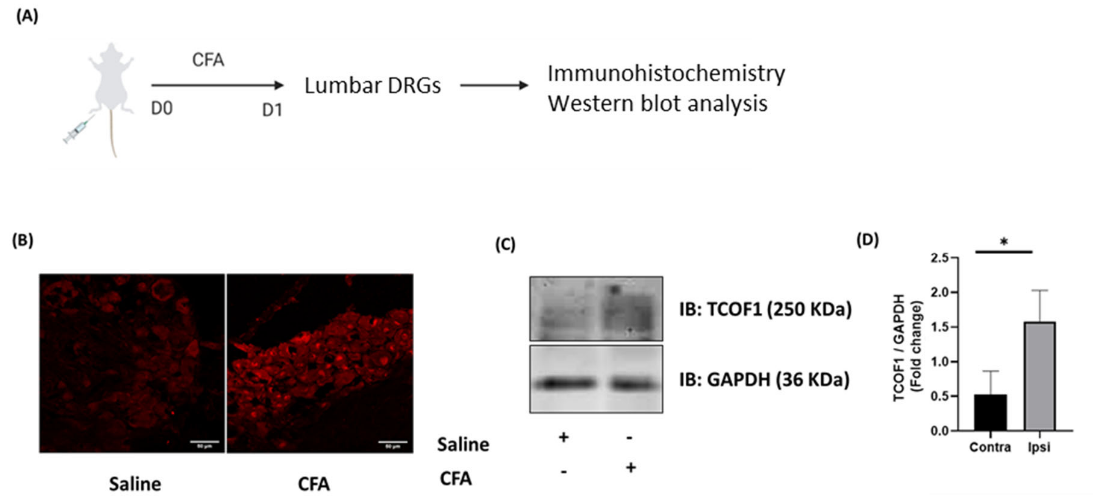


**Supplementary figure 1**: TCOF1 protein level is upregulated in lumbar DRGs from CFA-injected mice. (A) Schematic illustration of the CFA administration protocol. Mice received intraplantar CFA injections at day 0. Twenty-Four hours later, lumbar DRG (L4-L6) were harvested for immunohistochemistry and western blot analysis. (B) Representative confocal images of TCOF1 expression in DRG sections of ipsilateral lumbar DRGs following intraplantar injection with saline or CFA (Scale bar: 50 µm). (C) TCOF1 protein level was determined by Western blot in contralateral (black bar, n=4) or ipsilateral lumbar (grey bar, n=4) DRGs. (D) Bar chart representing the protein level of TCOF1 (normalized to GAPDH) illustrated in C. Statistical analysis was performed using Mann–Whitney U test; *P<0.05. N=4 experiments.

Vxssohphqwdu|#Wdeoh#4=#Surwhlqv#hqulfkhg#lq#ghwhfwlqj#zlwk#6<8T#DUUE5O\IS##

| Protein Name | Gene Name | Fold Change |
|---|---|---|
| Putative killer cell immunoglobulin-like receptor-like protein KIR3DX1 | KIR3DX1 | 10119 |
| Pleckstrin homology domain-containing family G member 4B | PLEKHG4B | 22.129 |
| Heterogeneous nuclear ribonucleoprotein U | HNRNPU | 12.059 |
| RNA-binding protein 39 | RBM39 | 10.502 |
| TIMELESS-interacting protein | TIPIN | 7.2743 |
| Semaphorin-6D | SEMA6D | 7.2578 |
| U2 small nuclear ribonucleoprotein A' | SNRPA1 | 6.7652 |
| rRNA 2'-O-methyltransferase fibrillarin | FBL | 5.4001 |
| Mitochondrial amidoxime reducing component 2 | MTARC2 | 5.0321 |
| Desmoplakin | DSP | 4.7156 |
| Splicing factor U2AF 35 kDa subunit | U2AF1 | 4.6847 |
| Peroxisomal acyl-coenzyme A oxidase 3 | ACOX3 | 4.6146 |
| Double-stranded RNA-specific adenosine deaminase | ADAR | 4.5461 |
| RNA-binding protein 14 | RBM14 | 4.3896 |
| Pre-mRNA-processing factor 40 homolog A | PRPF40A | 4.3845 |
| Granulocyte-macrophage colony-stimulating factor receptor subunit alpha | CSF2RA | 4.2261 |
| Histone H4 | HIST1H4A | 4.1101 |
| Dedicator of cytokinesis protein 10 | DOCK10 | 3.8544 |
| Zinc finger protein 90 homolog | ZFP90 | 3.8201 |

| | | |
|---|---|---|
| Zinc finger protein 184 | ZNF184 | 3.7112 |
| Cellular tumor antigen p53 | TP53 | 3.6778 |
| Beta-1-syntrophin | SNTB1 | 3.5513 |
| Matrin-3 | MATR3 | 3.4671 |
| U4/U6.U5 tri-snRNP-associated protein 1 | SART1 | 3.2183 |
| DNA replication licensing factor MCM7 | MCM7 | 3.1693 |
| Protein FAM107B | FAM107B | 2.9587 |
| Treacle protein | TCOF1 | 2.9354 |
| Myb-binding protein 1A | MYBBP1A | 2.7657 |
| Nucleolar and coiled-body phosphoprotein 1 | NOLC1 | 2.7034 |
| Junction plakoglobin | JUP | 2.6995 |
| Serine/arginine repetitive matrix protein 2 | SRRM2 | 2.6524 |
| Nucleolar transcription factor 1 | UBTF | 2.6395 |
| Nucleolar protein 56 | NOP56 | 2.6324 |
| Mediator of RNA polymerase II transcription subunit 12 | TNRC11;MED12 | 2.5634 |
| Poly [ADP-ribose] polymerase 1 | PARP1 | 2.4962 |
| DnaJ homolog subfamily C member 14 | DNAJC14 | 2.4796 |
| G patch domain-containing protein 4 | GPATCH4 | 2.4613 |
| ATP-binding cassette sub-family A member 8 | ABCA8 | 2.4598 |
| DNA-dependent protein kinase catalytic subunit | PRKDC | 2.4487 |
| Enhancer of rudimentary homolog | ERH | 2.4389 |
| Retinitis pigmentosa 9 protein | RP9 | 2.4284 |
| Collagen alpha-3(V) chain | COL5A3 | 2.414 |
| Multiple myeloma tumor-associated protein 2 | MMTAG2 | 2.3756 |
| Polycystic kidney disease 2-like 2 protein | PKD2L2 | 2.216 |
| Spliceosome RNA helicase DDX39B | DDX39B | 2.2018 |
| DNA topoisomerase 1;DNA topoisomerase I, mitochondrial | TOP1;TOP1MT | 2.1933 |
| Heterogeneous nuclear ribonucleoproteins A2/B1 | HNRNPA2B1 | 2.1842 |
| Apoptotic chromatin condensation inducer in the nucleus | ACIN1 | 2.1823 |
| Ribosomal L1 domain-containing protein 1 | RSL1D1 | 2.172 |
| Serine/arginine-rich splicing factor 7 | SRSF7 | 2.1663 |
| Lysine-specific histone demethylase 1A | KDM1A | 2.1599 |
| H/ACA ribonucleoprotein complex subunit 4 | DKC1 | 2.1594 |
| Non-histone chromosomal protein HMG-17 | HMGN2 | 2.1577 |
| Phosducin-like protein | PDCL | 2.1481 |
| Transcription activator BRG1 | SMARCA4 | 2.1194 |
| Obscurin | OBSCN | 2.1063 |
| Beta-arrestin-2 | ARRB2 | 2.0975 |
| Nucleophosmin | NPM1 | 2.0918 |
| U1 small nuclear ribonucleoprotein 70 kDa | SNRNP70 | 2.0792 |
| Mitochondrial import inner membrane translocase subunit Tim10 | TIMM10 | 2.0459 |

| | | |
|---|---|---|
| Heterogeneous nuclear ribonucleoprotein A3 | HNRNPA3 | 2.039 |

Vxssohphqwdu|#Wdeoh#5=#Surwhlqv#ghfuhdvlqj#zlwk#ZW#DUUE50\IS##

| Protein Name | Gene Name | Fold change |
|---|---|---|
| Mediator of RNA polymerase II transcription subunit 13-like | MED13L | 0.000046162 |
| Mirror-image polydactyly gene 1 protein | MIPOL1 | 0.00054549 |
| Protein Lines homolog | LINS | 0.0070436 |
| Adenomatous polyposis coli protein | APC | 0.01343 |
| Serine/threonine-protein kinase NIM1 | NIM1K | 0.020143 |
| Coiled-coil domain-containing protein 37 | CCDC37 | 0.020428 |
| Leukocyte surface antigen CD47 | CD47 | 0.025871 |
| Ras GTPase-activating protein 4 | RASA4 | 0.03178 |
| Eukaryotic translation initiation factor 3 subunit H | EIF3H | 0.033853 |
| High mobility group protein B1 | HMGB1 | 0.046925 |
| Beta-arrestin-2 | ARRB2 | 0.054871 |
| KRAB domain-containing protein 1 | KRBOX1 | 0.059237 |
| Mediator of RNA polymerase II transcription subunit 13-like | KCT2 | 0.082318 |
| Peptidyl-prolyl cis-trans isomerase NIMA-interacting 4 | PIN4 | 0.08355 |
| Islet amyloid polypeptide | IAPP | 0.089636 |
| Tenascin-X | TNXB | 0.1172 |
| Endothelial differentiation-related factor 1 | EDF1 | 0.12316 |
| Peptidyl-prolyl cis-trans isomerase B | PPIB | 0.1498 |
| Pleiotrophin | PTN | 0.16178 |
| High mobility group protein B2 | HMGB2 | 0.16984 |
| Bromodomain-containing protein 4 | BRD4 | 0.17341 |
| Radial spoke head protein 4 homolog A | RSPH4A | 0.20045 |
| Sulfotransferase 1C2 | SULT1C2 | 0.20169 |
| 40S ribosomal protein S9 | RPS9 | 0.2049 |
| Serine/threonine-protein kinase RIO1 | RIOK1 | 0.20925 |
| Axonemal dynein light chain domain-containing protein 1 | C1orf125 | 0.22575 |
| Leucine-rich repeat-containing protein 17 | LRRC17 | 0.26972 |
| Serine/arginine-rich splicing factor 7 | SRSF7 | 0.28439 |
| Histone H4 | HIST1H4A | 0.28585 |
| HIV Tat-specific factor 1 | HTATSF1 | 0.28676 |
| Titin | TTN | 0.30716 |
| 60S acidic ribosomal protein P0 | RPLP0 | 0.31348 |
| Ribosome-binding protein 1 | RRBP1 | 0.32688 |
| Zinc finger protein 174 | ZNF174 | 0.33538 |
| High mobility group protein B2 | HMGB2 | 0.34254 |
| V-type proton ATPase subunit G 2 | ATP6V1G2 | 0.35319 |

| | | |
|---|---|---|
| Protein DEK | DEK | 0.37908 |
| Gamma-adducin | ADD3 | 0.37968 |
| Tubulin alpha-1B chain | TUBA1B;TUBA4A | 0.37984 |
| Rho-related BTB domain-containing protein 2 | RHOBTB2 | 0.38706 |
| Elongation factor 1-alpha 1 | EEF1A1P5 | 0.39732 |
| Histone H2B | HIST1H2BN | 0.40223 |
| Coiled-coil domain-containing protein 124 | CCDC124 | 0.42408 |
| AFG3-like protein 2 | AFG3L2 | 0.44635 |
| Splicing factor U2AF 65 kDa subunit | U2AF2 | 0.44657 |
| E2/E3 hybrid ubiquitin-protein ligase UBE2O | UBE2O | 0.45298 |
| 60S ribosomal protein L7 | RPL7 | 0.45649 |
| Zinc finger CCCH domain-containing protein 15 | ZC3H15 | 0.47036 |
| Mitotic checkpoint serine/threonine-protein kinase BUB1 | BUB1 | 0.47521 |
| Chromodomain-helicase-DNA-binding protein 1 | CHD1 | 0.47626 |
| Alpha-enolase | ENO1 | 0.47727 |
| Activated RNA polymerase II transcriptional coactivator p15 | SUB1 | 0.48508 |
| 60S ribosomal protein L30 | RPL30 | 0.48755 |
| 40S ribosomal protein S5 | RPS5 | 0.49399 |
| Heterogeneous nuclear ribonucleoprotein A/B | HNRNPAB | 0.49705 |
| FACT complex subunit SPT16 | SUPT16H | 0.49764 |
| Protein phosphatase 1G | PPM1G | 0.49785 |

#
